# Clinical characteristics in patients with cervicogenic dizziness: A systematic review

**DOI:** 10.1002/hsr2.134

**Published:** 2019-07-26

**Authors:** Mari Kalland Knapstad, Stein Helge Glad Nordahl, Frederik Kragerud Goplen

**Affiliations:** ^1^ Norwegian National Advisory Unit on Vestibular Disorders, Department of Otorhinolaryngology and Head and Neck Surgery Haukeland University Hospital Bergen Norway; ^2^ Department of Clinical Medicine University of Bergen Bergen Norway

**Keywords:** dizziness, neck pain, proprioception, vertigo

## Abstract

**Background and aims:**

Cervicogenic dizziness (CD) is a clinical syndrome of dizziness associated with neck dysfunction. CD represents a considerable diagnostic challenge since dizziness and neck pain are common symptoms with complex and multifactorial etiologies. Both research and clinical work on CD is limited by the lack of accepted diagnostic criteria. The aim of this study was to review clinical studies on CD and to assess current evidence regarding the clinical characteristics of this syndrome.

**Methods:**

A comprehensive PubMed and MEDLINE search was conducted from the date of inception of the database, with the last search conducted in September 2018. Included studies had to contain operable diagnostic criteria as well as a comparison between patients considered to have CD and a clinical comparison group. Data extracted were clinical outcomes, diagnostic criteria, age, sex, and sample size. Studies were assessed for methodological quality using the Crowe Critical Appraisal Tool.

**Results:**

Out of 2161 screened studies, eight studies comprising 225 patients met the inclusion criteria. Studies were of low to acceptable methodological quality. The most frequent and consistent clinical characteristic in patients classified as having CD, compared with other populations, was reduced posturographic stability. The most consistent diagnostic criteria were based on the concurrence of neck pain with dizziness after exclusion of other possible reasons for dizziness.

**Conclusion:**

There are few studies examining clinical characteristics in patients with cervicogenic dizziness. Altered posturography appeared to be the only consistent characteristic used when distinguishing CD from other populations. Diagnostic criteria currently used in research are likely to have low specificity, since they rest on the exclusion of other causes rather than on positive distinctive features. More studies are needed to better understand the clinical interrelations between dizziness and neck pain.

## 1 INTRODUCTION

The clinical diagnosis of cervicogenic dizziness (CD) is commonly reserved for patients presenting with dizziness associated with neck dysfunction after all other potential causes for the dizziness have been excluded.^1^ However, the usefulness of this diagnostic approach in a clinical setting is limited for several reasons.

Dizziness is a common symptom that may arise from a great number of disorders.^2^, ^3^ In approaching the dizzy patient, it is essential to narrow down this number by assessing symptoms, their time course, and possible triggers. The word “dizziness,” in itself, is insufficient to qualify as a diagnostic criterion. Typical clinical symptoms of CD are suggested to consist of disorientation, lightheadedness, or disequilibrium accompanied by cervical pain, limited range of motion, and reduced balance.^4^, ^5^ In addition, a close temporal relationship between the dizziness and neck symptoms is considered important by some authors (Wrisley et al 2000). An *ex juvantibus* confirmation of the diagnosis—based on the resolution of dizziness after treatment of the neck disorder—has been proposed.^1^ However, clinical studies documenting the vestibular or extra‐vestibular symptoms, whether they be vertiginous or not, whether acute, episodic, or chronic, or triggered by specific activities or events, are needed. CD has several proposed causes, such as vascular or neurovascular.^6^ However, the most common theory is considering CD to be a disorder of neck proprioception.^1^, ^7^ Furman and Cass^7^ defined it as a “nonspecific sensation of altered orientation in space and disequilibrium originating from abnormal afferent activity from the neck.” Because of high demands of both stability and mobility, the cervical spine has a well‐developed proprioceptive system.^8^, ^9^, ^10^ Thus, the functional status of the neck should be examined, and the use of neck pain as a diagnostic marker of CD may, therefore, be inadequate. Thus, there is a need for clinical studies documenting neck function in patients with CD.

To date, there is no consensus on diagnostic criteria for CD. Several reviews have been published on the topic, but these have mainly focused on the theoretical basis for the diagnosis, eg, the abundance of muscle spindles in the deep cervical muscles,^10^ the close integration between cervical and vestibular afferents in the brain stem and cerebellum,^11^ and experimental studies on the effect of selective neck lesions or injection on balance and dizziness.^12^, ^13^, ^14^, ^15^ To the authors' knowledge, no systematic review exists of clinical studies on CD and how these patients differ from other relevant patient populations such as those with other diagnosis of dizziness, patients with neck pain, or even healthy controls. Identifying studies examining how CD patients differ from other populations would contribute to better understand the condition and guide future research.

The aim of this paper is to review clinical studies on CD and to assess current evidence regarding the clinical characteristics of this syndrome. A secondary aim was to examine and compare the diagnostic criteria that were used in the included studies.

## 2 METHODS

### 2.1 Study design

This systematic review adhered to the guidelines outlined in the Preferred Reporting Items for Systematic Reviews and Meta‐Analysis (PRISMA) Statement.^17^

### 2.2 Search strategy

A comprehensive literature search was performed through PubMed and MEDLINE from the inception of the database to September 2018 (last search date: 9th of September 2018). The search terms were used as mesh terms or text words and were adjusted for the different databases. The search terms and the full search strategy are available in Appendix S1. Each step in the screening process was performed by two reviewers independently (MKK and FKG). References from included papers were screened by the reviewers for potentially relevant studies not captured by the electronic search.

### 2.3 Eligibility criteria

This review was restricted to published, peer‐reviewed original studies. Unpublished studies, case reports, editorials, reviews, and conference abstracts were not included. The search was restricted to articles written in English. We included original studies on patients with CD because of allegedly altered neck proprioception, comparing their clinical characteristics to those of other populations. Thus, for inclusion, the study had to contain a reference group, either with another diagnosis or healthy controls, for comparison. To assure higher comparability between studies, included studies had to state whether or not other causes of dizziness had been ruled out. This included other causes of alleged CD such as neurovascular or vascular disorders. In addition, the diagnostic process or criteria had to be accounted for. Studies were excluded if the study population (CD) was composed of patients suffering from other confirmed diseases that could explain their symptoms. For readability and consistency, this review uses the term CD, although some of the included papers have used slightly different names for the same condition (Table 1).

**Table 1:** Included studies in the review (n = 8)

Article	Type of Study	Diagnostic Criteria	Patients with Cervicogenic Dizziness	Reference Group	Outcome Measures	Main Findings	CCAT Score
Reid, S. A., Callister, R., Katekar, M. G., & Treleaven, J. M. (2017)	Case‐control	Stiff and/or painful neck, dizziness described as “unsteadiness” triggered by neck movement, Palpable upper cervical joint dysfunction on assessment by an experienced musculoskeletal physiotherapist. Other causes excluded by assessment by neuro‐otologist	Patients with cervicogenic dizziness (n = 86; age: 66; sex: 43 females, 43 males).	Patients with dizziness of other, non‐cervicogenic causes (n = 86; mean age: 64; sex: 55 females, 31 males).	DHI	Questions 1 (Does looking up increase your problem?), 9 (Because of your problem, are you afraid to leave your home without having someone accompany you?), and 11 (Do quick movements of your head increase your problem) on DHI were most discriminatory to cervicogenic dizziness compared with general dizziness. The optimal threshold on these scores were <9 for cervicogenic dizziness.	26
Karlberg, M., Johansson, R., Magnusson, M., & Fransson, P. A. (1996)	Cross‐sectional	Neck pain and concomitant complaints of dizziness or vertigo. Other causes excluded.	Patients with vertigo of suspected cervical origin (n = 16; age: 38; sex: 14 females, 2 males.)	Patients with vestibular neuritis (*n* = 18; mean age: 49; sex: 10 females, 8 females). Controls (n = 17. Mean age: 40. Sex: 9 females, 8 males.).	Posturographic measurement of postural responses to vibratory stimulation of the calf muscles	Patients with cervicogenic dizziness were distinguished both from controls and VN with regard to disturbed postural control. Both in the “eyes open” and “eyes closed” conditions; patients with suspected cervical vertigo were characterized by significantly lower values for stiffness and significantly higher values of damping compared with healthy controls and significant lower values for stiffness than the VN patients for any of the individual parameters under any test conditions.	28
L'Heureux‐Lebeau, B. Godbout, A. Berbiche, D.& Saliba, I. (2014)	Case‐control	Neck pain associated with dizziness Cervical pain, trauma/or disease If from traumatic origin, temporal proximity between the onset of dizziness and the neck injury. Other causes excluded.	Patients with cervicogenic dizziness (n = 25; sex: 22 female, 3 male; age: 49.12 [10.21]).	Patients with benign paroxysmal positional vertigo (n = 25; sex: 20 female, 5 male; mean age: 57.28 [16.17]).	Smooth pursuit Neck torsion test Cervical torsion test Cervical relocation test DHI State trait anxiety Inventory Dizziness characteristics Neck pain	There was a significant difference in mean cervical joint position error, and videonystagmography showed differences in the cervical torsion test between the two groups. No difference in DHI or anxiety was observed. There was a difference in sensorimotor disturbances between the two groups, particularly in the control of head and eye movement and cervical proprioception. Patients with cervicogenic dizziness were more likely to have sensation of drunkenness/lightheadedness, pain induced during examination of upper cervical vertebra, joint position error of 4.5° during cervical relocation test, and exhibit more than 2° per second nystagmus during cervical rotation test.	25
Karlberg, M. Magnusson, M. Malmstrom, E. M., Meler, A., & Moritz, U. (1996)	Prospective randomized, controlled trial	Recent onset of neck pain and simultaneous complaints of dizziness or vertigo Extracervical causes of dizziness excluded.	Patients with dizziness of suspected cervical origin (n = 17; sex: 15 female, 2 male; age: 39)	Healthy controls (n = 17; sex: 15 female, 2 male; mean age: 35)	Posturography	Patients with cervicogenic dizziness had impaired postural performance compared with healthy controls in all posturographic conditions.	23
Grande‐Alonso, M. Moral Saiz, B. Minguez Zuazo, A. Lerma Lara, S. & La Touche, R. (2016)	Cross‐sectional	Neck pain on visual analogue scale Neck pain according to the Neck Disability Index Dizziness associated with pain, movement, rigidity, or certain neck positions Duration of neck pain and dizziness >3 mo Age 18‐65 years	Cervicogenic dizziness (n = 20; sex: 18 female, 2 male; age: 36.5 [11.03]).	Asymptomatic healthy controls (n = 22; sex: 15 female; 7 male; mean age: 35.2 [10.03])	VOR activity Postural control TSK‐11 HADS anxiety HADS depression	There was no difference in VOR activity between patients with cervicogenic dizziness and asymptomatic subjects. There were differences with a medium‐to‐large effect size in variables related to proprioception and visual information integration. There was a difference in TSK‐11 and HADS anxiety and HADS depression.	24
Yahia, A. Ghroubi, S. Jribi, S. Malla, J. Baklouti, S. Ghorbel, A. & Elleuch, M. H. (2009)	Cross‐sectional	Chronic neck pain (>3 mo (in presence or absence of vertigo) linked to cervical arthritis or minor intervertebral disorders Excluded patients with a history of cervical spine trauma or surgery or those with abnormal results in ear, nose, and throat examinations (vestibular damage), ophthalmological test (vision disorders), and/or neurological assessment (sensorimotor or coordination impairments).	Chronic neck pain patients with vertigo (G1) (n = 32; age: 48.15; sex: 68.7% female).	Neck pain (G2) (n = 30; mean age: 47.1; sex: 76.66 % female). Healthy (G3) (n = 30; age: 47.13; sex: 83.33 % female).	VAS CROM Neck‐related headache Static and dynamic posturography	The mean neck pain intensity on a VAS was 6.65 out of 10 in G1 and 4.03 in G2. Cervical spine mobility was significantly lower in G1 than in G2 and G3. Neck‐related headache was more frequent in G1 than in G2 (65.5% vs 40%, respectively). Balance abnormalities were found more frequently in G1 than in G2 or G3. Static and dynamic posturographic assessments (under “eyes open” and “eyes shut” conditions) revealed significant abnormalities in statokinetic parameters in G1.	14
Alund, M., Ledin, T., Odkvist, L., & Larsson, S. E. (1993)	Cross‐sectional	Localized neck pain and stiffness for more than one year Long‐lasting general neck pain as well as vertigo and/or unsteadiness Central and peripheral vestibular abnormalities excluded	Patients with suspected cervical vertigo (n = 15; age: 48; sex: 12 females, 3 males).	Neck pain (n = 10; mean age: 47; sex: 6 female, 4 male.). Healthy (n = 15; age and sex matched).	VAS CROM Dynamic posturography	There was no difference in VAS between cervicogenic vertigo and the neck pain group and no difference between groups in neck range of motion. Patients with cervicogenic vertigo had significant lower mean equilibrium scores with head in neutral position, left rotation, and right lateral rotation compared with controls. Patients with cervicogenic dizziness had lower equilibrium score when examined in the position most prone to elicit vertigo/unsteadiness compared with neck patients.	16
Heikkila, H. Johansson, M. & Wenngren, B. I. (2000)	Single subject	Patients with complaints of dizziness or vertigo of suspected cervical origin. Excluded if there was a possibility of extracervical causes, older than 55, vertigo persisting in less than 3 mo or with a history of central nervous system diseases or trauma, ear disease, arteriosclerotic, or rheumatoid arthritis.	Patients with complaints of dizziness or vertigo of suspected cervical origin (n = 14; sex: 8 female, 6 men; age: 36).	Healthy volunteers (n = 39; sex: 24 female, mean age: 35).	Kinesthetic sensibility test	Significant differences in relocation success was found in all directions in flexion, extension, and rotation between groups.	17

Abbreviations: Age: reported as mean; CROM, cervical range of motion; DHI, Dizziness Handicap Inventory; HADS, hospital anxiety and depression scale; TSK, Tampa Scale for Kinesophobia; VAS, visual analog scale; VN, vestibularis neuritis; VOR, vestibular ocular reflex.

### 2.4 Study selection

**Figure 1:**
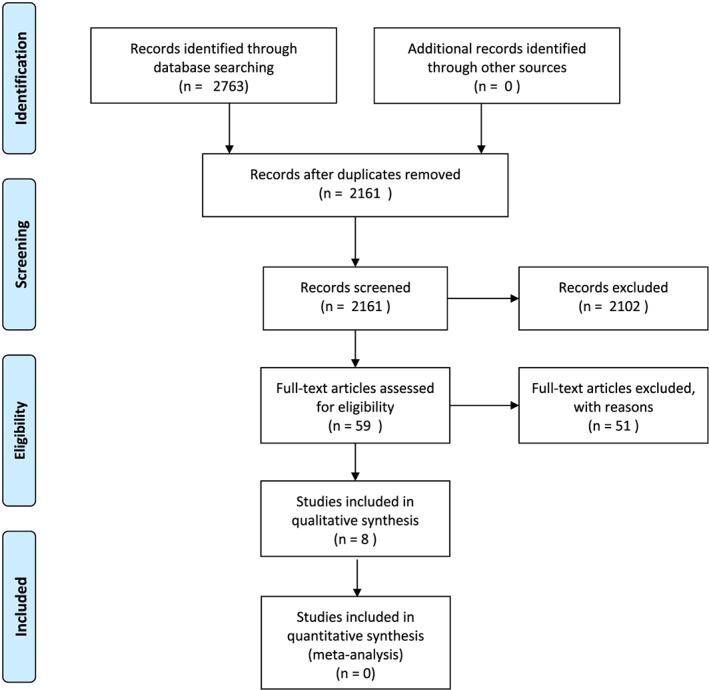
Illustration of the study selection process with the PRISMA 2009 Flow Diagram

All titles and abstracts were screened by the two reviewers after duplicates were discarded and irrelevant citations were removed. Full text versions of eligible articles were evaluated by the two reviewers to determine inclusion. Any disagreements were resolved through discussion among reviewers. The process was facilitated by the use of the Rayyan systematic review web application,^16^ which allows for blinding in each step of the process. The PRISMA 2009 Flow Diagram (Figure 1)^17^ illustrates the selection process of the studies.

### 2.5 Data extraction process

The following data were extracted, compared, and compiled in a spreadsheet by both reviewers: population (age, sex, and sample size), study design, diagnostic criteria, and clinical findings compared with other diagnosis/healthy controls. The two reviewers compared the entered data and corrected missing entries.

### 2.6 Assessment of methodological quality

Because of the heterogeneous nature of the studies with regard to design and outcome measures, quality of data, and study design, a meta‐analysis was not appropriate for this review. Thus, a quality analysis of the included studies was performed. The methodological quality of the studies was assessed using the Crowe Critical Appraisal Tool version 1.4 (CCAT), which allows for a variety of research designs to be evaluated using the same tool.^18^ This tool consists of nine categories. The first eight categories have a score range from 0 to 5. The ninth category states the total sum from the previous eight categories, which can range from 0 to 40, where a higher score indicates higher quality.

### 2.7 Ethical Approval

Ethical approval was not required for this systematic review.

## 3 RESULTS

### 3.1 Search

The search resulted in 2161 articles, after removing duplicates. After screening titles and abstracts for irrelevant citations, we identified 59 articles, which were assessed in full text. No additional articles were found when screening reference lists. Fifty‐one studies did not meet the inclusion criteria and were excluded from this review. See Appendix S2 for a list of excluded studies with reasons for exclusion. A total of eight studies met the inclusion criteria. The selection process is shown in Figure 1. The eight included studies comprised four cross‐sectional studies,^19^, ^20^, ^21^, ^22^ one prospective study,^23^ two case‐control studies,^24^, ^25^ and one single‐subject design study.^26^ The included studies comprised a total of 225 patients classified as CD, with group sizes ranging from n = 14 to 86. Patients were compared with healthy controls (n = 140) in five studies,^19^, ^21^, ^22^, ^23^, ^26^ to patients with BBPV (n = 25) in one study,^24^ to patients with general dizziness (n = 86) in one study,^25^ to patients with vestibular neuritis (n = 18) in one study,^21^ and to patients with only neck pain (n = 40) in two studies.^19^, ^22^ Most studies included more women (n = 136) than men (n = 89), with the percentage of women ranging from 42 % to 87 %. The age of the CD patients ranged from 36 to 66 years. The included studies, with methodological quality assessment, are shown in Table 1.

### 3.2 Clinical findings

#### 3.2.1 Posturography

A total of five studies included posturography. One of the studies found that the posturographic response to vibratory stimulation of the calf muscles could distinguish patients with vertigo of suspected cervical origin from patients with vestibular neuronitis and healthy controls.^21^ Two of the studies found that patients with CD had reduced postural control compared with both patients with only neck pain and healthy controls.^19^ The last two studies found reduced postural control in CD patients compared with healthy controls.^20^, ^23^

#### 3.2.2 Cervical proprioception measured by relocation tests

Two studies examined cervical proprioception using relocation tests. These tests use a laser placed on the patient's forehead to measure the overshoot/undershoot when patients attempt to move the head back to a neutral position (straight ahead) after different head turns.^24^, ^26^ L'Heureux‐Lebeau et al.^24^ reported that patients classified with CD had a higher positioning error compared with patients with BPPV. Heikkila et al.^26^ reported higher relocation errors after cervical flexion, extension, and rotation in patients classified with CD compared with healthy controls.^26^

#### 3.2.3 Cervical range of motion

Cervical range of motion (CROM) was examined in two studies, with different measurements methods.^19^ Yahia et al.^22^ found that patients with chronic neck pain and vertigo had significantly lower CROM (measured in centimeters from chin to sternum, chin to acromion, and earlobe to acromion) compared with both patients with only chronic neck pain and healthy controls. Alund et al.^19^ found no difference in CROM (measured with a three‐dimensional electrogonimetric equipment) between patients with suspected CD, neck pain, and healthy controls.

#### 3.2.4 Symptom duration

Two of the studies reported duration of dizziness. In one of the studies, the patients with CD had longer duration of dizziness (81 months) compared with patients with general dizziness (23 months).^25^ In the other, the patients with CD exhibited shorter dizziness duration (30 months) compared with patients with BPPV (38 months).^24^

#### 3.2.5 Neck pain

Neck pain was examined in three studies. L'Heureux‐Lebeau et al.^24^ found more frequent neck pain in patients classified as having CD compared with patients with BPPV. Alund et al.^19^ found no difference in neck pain between patients with CD and patients with only neck pain. The other study, that of Yahia et al,^22^ found that chronic neck pain patients with vertigo scored significantly higher on neck pain compared with chronic neck pain patients without vertigo.

#### 3.2.6 Psychometric measures

L'Heureux‐Lebeau et al.^24^ found no difference in anxiety or dizziness handicap between patient with CD and those with BPPV, using the Dizziness Handicap Inventory and State‐Trait Anxiety Inventory. Grande‐Alonso et al.^20^ found that patients with CD had higher fear of movement and higher anxiety and depression levels than asymptomatic individuals, as measured by the Tampa Scale for Kinesophobia and Hospital Anxiety and Depression Scale.

#### 3.2.7 Dizziness characteristics and triggers

Only one study examined differences in dizziness characteristics between patients with CD and other dizziness diagnoses. The study found that patients with CD were more likely to have a sensation of drunkenness/lightheadedness^24^ compared with patients with BPPV. Patients with BPPV were more likely to experience rotatory vertigo. The CD group was more likely to report cervical movement as a precipitating factor. There were no differences in self‐reported imbalance, dizziness, lightheadedness, floating sensation, sway sensation, nausea, falls, or dizziness frequency between the two groups. Reid et al.^25^ found that Questions 1 (Does looking up increase your problem?), 9 (Because of your problem, are you afraid to leave your home without having someone accompany you?), and 11 (Does quick movement of your head increase your problem) of the Dizziness Handicap Inventory allowed to better classify patients as having CD compared with general dizziness.

#### 3.2.8 Headache

One of the included studies^22^ found that patients with chronic neck pain and vertigo had more neck‐related headaches compared with patients with only chronic neck pain.

#### 3.2.9 Smooth pursuit, nystagmus during neck torsion, video head impulse test (vHIT)

L'Heureux‐Lebeau et al.^24^ reported that patients with CD were more likely than patients with BPPV to have a positive smooth pursuit neck torsion test as well as nystagmus elicited by neck torsion (2° per second or more). However, the criteria for the former test were not specified. Grande‐Alonso et al. (2016) reported no difference in vHIT responses between patients with CD and asymptomatic individuals.

### 3.3 Diagnostic criteria

#### 3.3.1 Coexistence of dizziness and neck pain

All but one^26^ of the included studies had the coexistence of neck pain and dizziness as an explicit diagnostic criterion. In Heikkila et al.,^26^ neck pain was implicated in the criterion “dizziness or vertigo of suspected cervical origin.”

#### 3.3.2 Vestibular symptoms, triggers, and aggravating factors

Most of the included studies did not specify particular dizziness symptoms as criteria for classifying patients as CD. However, one study^25^ included dizziness “described as unsteadiness triggered by neck movement” as a criterion. Another study included dizziness “associated with pain, movement rigidity, or certain neck positions” as a criterion.^20^

#### 3.3.3 Timing and duration of neck symptoms and dizziness

Four of the included studies specified duration of symptoms in the diagnostic criteria. One study reported that the patients had to have “recent onset” of and simultaneous complaint of dizziness or vertigo.^21^ Another reported that the duration of both neck pain and dizziness had to be longer than 3 months.^20^ Yahia et al.^22^ used chronic neck pain of more than 3‐month duration as a criterion. Alund et al.^19^ chose neck pain and stiffness for more than one year as a criterion. The criteria for dizziness were only reported as “long‐lasting.” Finally, one study added that if the neck pain had a traumatic origin, there needed to be a temporal proximity between the onset of dizziness and the neck injury.^24^

#### 3.3.4 Neck examination

Two studies included decreased neck mobility in the diagnostic criteria.^19^ Reid et al.^25^ reported stiff and/or painful neck as one of their criteria, whereas Alund et al.^19^ mentioned “localized neck pain and stiffness.” Reid et al.^25^ additionally required “palpable upper cervical spine dysfunction” assessed by an experienced physical therapist.

#### 3.3.5 Other causes excluded

All studies reported exclusion of causes of dizziness/vertigo, such as vestibular and central. The studies described in detail the method and examination used for ruling out patients with other causes of dizziness or vertigo, except for one.^20^ However, this study noted that presence of an otorhinolaryngological diagnosis of central or peripheral vertigo would exclude the patient from their study.

### 3.4 Methodical quality of the studies

The studies were given CCAT scores ranging from 14 to 28, indicating low to acceptable methodical quality. Common limitations in the included studies were insufficient information on sampling methods, insufficient sample size justification, insufficient information on ethical matters, and limitations related to statistical analysis.

## 4 DISCUSSION

This review identified eight original studies comparing patients with CD with groups of patients either suffering from other established and well‐defined conditions or healthy controls. Based on CCAT scores, the studies were of low to acceptable methodological quality. Pooling of the results was not possible since outcomes varied. Nevertheless, the studies shed some light on current opinions on CD.

### 4.1 Clinical findings

Although the International Classification of Vestibular Disorders distinguishes between vertigo and dizziness,^27^ and some consider it unlikely that disorders of neck proprioception should be associated with illusory perceptions of self‐motion such as spinning vertigo,^28^ only one study in this review^25^ required the a priori exclusion of patients with vertigo, stressing that dizziness should be described as “unsteadiness.” This follows the definition by Furman & Cass,^7^ where patients with CD are more likely to have a “nonspecific sensation” of dizziness, in contrast to patients with BPPV or those with other types of vestibular disorders, where the dizziness is usually reported as rotatory.^7^ However, one of the included studies^21^ found that seven out of 16 patients with dizziness of suspected cervical origin reported vertigo defined as a sensation of movement. L'Heureux‐Lebeau et al.^24^ found that 32% of patients with CD reported a rotatory sensation compared with 76% in a group with BPPV. In this study, most patients reported a sensation of “drunkenness” (92%) or imbalance (76%). Admittedly, one should not rely solely on the description of vestibular symptoms in making a diagnosis, since patients have difficulties reporting vestibular symptoms in a consistent way.^29^ However, a strong sensation of spinning vertigo should clearly lead to the suspicion of extracervical causes and probably also to the exclusion of CD as long as objective tests are unavailable to confirm this diagnosis.

The onset and time course of CD were not addressed specifically in any of the studies. While vestibular disorders like vestibular neuritis, BPPV, and Menière's disease are usually distinguished by an acute onset, dizziness caused by degenerative neck disorders would be expected to develop gradually. One of the included studies found that the average patient reported daily symptoms (mean score 4 on a frequency scale from 0 to 4).^21^ L'Heureux‐Lebeau et al.^24^ found that a large group (40%) reported attacks of a few seconds duration, while 32% reported constant dizziness, indicating a variable time course, although most patients had dizziness every day (76%). Compared with patients with BPPV, patients with CD more often reported aggravation of dizziness because of cervical pain, fatigue, anxiety, stress, and to “any neck movements.” Several of these factors also aggravate symptoms in patients with persistent postural‐perceptual dizziness,^30^ but some distinction should be possible because of the sensitivity of the latter group to visual and motion stimuli. The study by Reid et al.^25^ found that patients with CD were more likely to report aggravation of symptoms when looking up or during quick head movements than patients with dizziness of other causes. This seems reasonable based on the suspected pathophysiology of CD decreasing or altering proprioceptive feedback from the neck.^1^, ^7^ However, looking up and moving the head quickly also aggravates symptoms in patients with vestibular disorders, such as BPPV or vestibular neuritis. In addition, a way to distinguish peripheral vestibular lesions from nonvestibular causes of dizziness is by examining the vestibulo‐ocular reflex in response to high‐velocity head movements (eg, the head impulse test). These triggers can, therefore, hardly be considered diagnostic.

Based on the present studies, CD would be expected to cause vestibular symptoms of gradual onset and present on a daily basis, aggravated by neck pain and be related to any neck movements rather than to specific head positions.

Most of the studies focused on identifying objective signs in the patients with CD, such as abnormal postural sway during platform posturography or increased positioning errors during cervical relocation tests, with posturography as the most consistent finding. Even though CD is thought to be associated with limited CROM,^5^ the results found in this review were contradictory. L'Heureux‐Lebeau et al.^24^ reported finding nystagmus induced by neck torsion as well as pathology on the smooth pursuit neck torsion test; however, criteria for the latter finding were not specified. Compared with patients with BPPV, patients with CD were consistently sensitive to induced cervical pain during physical examination, particularly at the level of C3–C4. It seems reasonable to include a physical examination of neck tenderness and mobility in the diagnosis of CD, and because of the importance of neck proprioception to postural balance, quantitative measurements of posture and gait, particularly during dynamic conditions, might reveal diagnostically relevant information. However, because of the scarcity of data and the differences in outcome measures, more studies are needed before any conclusions can be made as to the usefulness of posturographic or cervical relocation tests in the diagnosis of CD.

### 4.2 Diagnostic criteria for cervicogenic dizziness

The diagnostic criteria used in the reviewed studies were predefined by the authors, and because of the lack of a diagnostic “gold standard,” their validity cannot be determined. The criteria of CD was, in most studies, based on the patient simultaneously reporting neck pain and dizziness as well as the exclusion of other neurological or neuro‐otological disorders. The distinction between vertigo and dizziness was not considered essential for the diagnosis in most of the reviewed studies. One study specified that the dizziness should be described as “unsteadiness”,^25^ while another required vertigo defined as an “erroneous impression of the movement of objects relative to the subject or the movement of the subject relative to his/her environment.”

Neck stiffness or rigidity was not usually required for the diagnosis but mentioned in the inclusion criteria of three studies.^19^, ^25^ The same was the case with localized tenderness in the neck, which was mentioned in two studies.^19^, ^25^ Positive objective signs were usually not considered necessary, except for one study^25^ that required “palpable upper cervical spine dysfunction” assessed by an experienced physiotherapist. Yahia et al.^22^ included patients with cervical arthritis or minor intervertebral disorders on standard cervical X‐ray imaging.

Symptom duration varied widely in the reviewed studies. Karlberg et al.^21^ required a recent onset of neck pain and simultaneous complaints of dizziness or vertigo. This may be reasonable simply because the patients' memory of the temporal relationship between the two symptoms would be more reliable. Conversely, long symptom duration may increase the likelihood of other comorbidities entering the equation, eg, functional disorders or dysfunction related to psychosocial consequences of long‐lasting disease. However, several authors had long‐lasting symptoms as a criteria. Heikkila et al.^26^ excluded patients with vertigo persisting for less than 3 months. Grande‐Alonso et al.^20^ required a duration of neck pain and dizziness for more than 3 months. Yahia et al.^22^ specified chronic neck pain for more than 3 months, while Alund et al.^19^ included patients with localized neck pain and stiffness for more than 1 year.

A specific time course and triggers of vestibular symptoms were not required for the diagnosis by most authors. Reid et al.^25^ included patients with dizziness described as unsteadiness triggered by neck movement. Grande‐Alonso et al.^20^ required dizziness associated with pain, movement, rigidity, or certain neck positions.

It has been argued that the diagnosis of CD may be mainly of exclusive academic interest, since the treatment is often the same as for patients with cervical pain syndrome.^28^ However, a correct diagnosis will always be clinically meaningful in guiding the treatment and in reassuring the patient that an explanation for their distressing symptoms has been found. Lastly, a conclusive diagnosis could save both the patients and the health care system from the consequences of unnecessary diagnostic and therapeutic procedures.

## 5 LIMITATIONS

The review was limited to studies reported in English. Because of the low number, varying outcomes, and the low to moderate methodological quality of the included studies, pooling of the data was not possible, and firm conclusions as to the nature and clinical characteristics of CD cannot be made. The review reflects diagnostic criteria in studies that met the inclusion criteria and is not representative of all studies on CD. However, the inclusion criteria were not likely to exclude valuable clinical studies on CD, stating merely that included studies should contain operable diagnostic criteria as well as a comparison group. The inclusion of studies comparing patients with CD with healthy controls makes the review somewhat heterogenic. However, with the general lack of clinical studies on CD, we found that comparisons with healthy controls would also contribute to the limited knowledge within the area. The review provides an overview of the current understanding of CD as reflected by existing clinical studies. However, it may be considered a limitation that the studies in this review were published from 1993 to 2017, with half of them being more than 9 years old. This highlights the need for further studies within this field.

## 6 CONCLUSIONS AND IMPLICATIONS

Studies comparing the clinical characteristics of patients with CD with other populations are few and of low to acceptable methodological quality. There is some evidence that patients with CD may have altered postural balance on platform posturography compared with patients with other diagnoses or healthy controls. Larger and more robust studies are needed to corroborate these findings and to establish the clinical syndrome of CD and whether it is indeed an independent and separate condition from other well‐established ones. Diagnostic criteria differed between studies and were mostly based on the coexistence of neck pain with dizziness and the exclusion of other neurological and neuro‐otological causes. Thus, the sensitivity and specificity of the criteria are likely to be low. As this review revealed significant differences in methodical and experimental approaches, this should be considered when designing future studies, making comparison between studies more feasible.

## CONFLICTS OF INTEREST

The authors declare that they have no competing interests.

## AUTHOR CONTRIBUTIONS

Conceptualization: Mari Kalland Knapstad, Frederik Kragerud Goplen

Formal analysis: Mari Kalland Knapstad, Frederik Kragerud Goplen

Writing—original draft preparation: Mari Kalland Knapstad

Writing—review and editing: Mari Kalland Knapstad, Frederik Kragerud Goplen, Stein Helge Glad Nordahl

All authors have read and approved the final version of the manuscript. Mari Kalland Knapstad had full access to all of the data in this study and takes complete responsibility for the integrity of the data and the accuracy of the data analysis.

## TRANSPARENCY STATEMENT

The lead author (MKK) affirms that this manuscript is an honest, accurate, and transparent account of the study being reported; that no important aspects of the study have been omitted; and that any discrepancies from the study as planned have been explained.

## DATA AVAILABILITY

The authors confirm that the data supporting the findings of this study are available within the article and/or its supplementary materials.

## FUNDING INFORMATION

The study was supported by the University of Bergen. The funder had no role in study design; collection, analysis, and interpretation of data; writing of the report; or the decision to submit the report for publication.

## Supporting information

Data S1: Supplementary Information

Data S2: Supplementary Information
