# Supplementary material for: Clinical characteristics in patients with cervicogenic dizziness: A systematic review
Source: Health Sci Rep. 2019 Jul 26;2(9):e134. doi: 10.1002/hsr2.134 (PMC6784794; doi:10.1002/hsr2.134)
Supplement: Supplementary file 1 — Data S1: Supplementary Information [file HSR2-2-e134-s001.docx]

**Appendix 1.**

Search strategy

Database: Ovid MEDLINE(R) Epub Ahead of Print, In-Process & Other Non-Indexed Citations, Ovid MEDLINE(R) Daily and Ovid MEDLINE(R) <1946 to Present>

--------------------------------------------------------------------------------

1 exp Vertigo/

2 Dizziness/

3 (vertigo or dizziness or dizzy).ti,ab,kw.

4 1 or 2 or 3 *= search on vertigo or synonyms*

5 Neck Pain/ *= search on Neck pain*

6 Chronic Pain/

7 Pain/

8 pain.ti,ab,kw.

9 6 or 7 or 8 *= search on pain*

10 Neck/

11 (cervical or cervicogenic or neck).ti,ab,kw.

12 10 or 11 *= search on neck*

13 9 and 12 *= combination of neck and pain= gives articles about neck pain*

14 5 or 13 *= articles about neck pain either based on subject headings or free text words*

15 4 and 14 *= combines vertigo and neck pain*

16 ((cervicogenic or cervical) adj2 (vertigo or dizziness)).ti,ab,kw. *= an additional search on textwords which combines vertigo with the words cercicogenic/cervical directly*

17 15 or 16 *= all search results on vertigo and neck pain*

*Explanations for Ovid databases*

*/ = subject heading from the controlled vocabulary*

*exp = Expands the search results of terms entered and include all of its narrower, more specific subject headings*

*ti,ab,kw. = search for free text words in title (ti), abstract (ab) , authors keywords (kw)*

Same rationale goes for the Embase search.

Database: Ovid Embase

Search on subject headings and free text words for: vertigo/dizziness and neck pain

<1946 to Present>

--------------------------------------------------------------------------------

1 exp vertigo/

2 dizziness/

3 (vertigo or dizziness or dizzy).ti,ab,kw.

4 1 or 2 or 3

5 neck pain/

6 chronic pain/

7 pain/

8 pain.ti,ab,kw.

9 6 or 7 or 8

10 neck/ or neck muscle/

11 (cervical or cervicogenic or neck).ti,ab,kw.

12 10 or 11

13 9 and 12

14 5 or 13

15 4 and 14

16 ((cervicogenic or cervical) adj2 (vertigo or dizziness)).ti,ab,kw.

17 15 or 16

18 limit 17 to conference abstract

19 17 not 18

Search on subject headings and free text words for: vertigo/dizziness and neck pain
