# Supplementary material for: Clinical characteristics in patients with cervicogenic dizziness: A systematic review
Source: Health Sci Rep. 2019 Jul 26;2(9):e134. doi: 10.1002/hsr2.134 (PMC6784794; doi:10.1002/hsr2.134)
Supplement: Supplementary file 2 — Data S2: Supplementary Information [file HSR2-2-e134-s002.doc]

**Appendix II. Excluded studies.**

| **Study** | **Reason for exclusion** |
| --- | --- |
| Anonymous, 1975 | Not possible to retrieve |
| Baron, 2011 | Wrong population * |
| Bartual, 1989 | No reference group |
| Bittar 2017 | Study design: Case report |
| Bolton, 1992 | Study design: Review |
| Borg-Stein, 2001 | Study design: Review |
| Bracher, 2000 | No reference group |
| Brandt, 2001 | Study design: Review |
| Brandt, 2016 | Study design: Case report |
| Chang, 2014 | No exclusion of other possible reasons for dizziness |
| Chen, 2015 | Wrong population * |
| Chen, 2002 | No reference group |
| Endo, 2008 | Wrong population * |
| Fitz – Ritson, 1991 | No reference group |
| Gu, 2016 | No reference group |
| Hou, 2017 | Study design: Systematic review |
| Humphrey, 2002 | No exclusion of other possible reasons for dizziness |
| Humphrey, 2013 | No exclusion of other possible reasons for dizziness |
| Iwasa, 1983 | No available in English |
| Jaroshevskyi, 2017 | No reference group |
| Johnston, 2017 | Wrong population * |
| Jongkees, 1969 | Study design: Review |
| Krabak, 2000 | No reference group |
| Li, 2013 | No reference group |
| Liu, 2017 | Wrong population * |
| Ma, 2015 | No reference group |
| Magnusson, 2016 | Study design: Review |
| Malmström. 2007 | No reference group |
| Michels, 2007 | No report of any clinical characteristics |
| Minguez – Zuazo. 2016 | No reference group |
| Morinaka. 2009 | No reference group |
| Moustafa, 2017 | No reference group |
| Norre, 1987 | Wrong population * |
| Nwaorgu, 2003 | No reference group |
| Okubo, 1973 | Not available in English |
| Reid, 2012 | Study design: Study protocol |
| Reid, 2014 | No reference group |
| Reid, 2015 | No reference group |
| Ren, 2014 | No reference group |
| Reneker, 2015 | Study design: Consensus report |
| Ryan, 1955 | Study design: Case report |
| Shum. 2017 | No reference group |
| Tjell, 1998 | Wrong population * |
| Van Leeuwen. 2017 | Wrong population * |
| Wakayama, 1973 | Not available in English |
| Wing, 1974 | No reference group |
| Wu, 2002 | No reference group |
| Xiaoxiang, 2006 | No reference group |
| Yacovino, 2013 | Study design: Review |
| Yin, 2016 | No reference group |
| Zeng, 2003 | Not available in English |
| * Not cervicogenic dizziness due to altered afferent information to the central nervous system | |

, A. (1975). Cervical vertigo. *Annals of Otology, Rhinology & Laryngology, 84*(2), 260-261.

Baron, E. P., Cherian, N., & Tepper, S. J. (2011). Role of greater occipital nerve blocks and trigger point injections for patients with dizziness and headache. *Neurologist, 17*(6), 312-317.

Bartual, J., Vidal, J., Magro, E., & Roquette, J. (1989). Effects of cinnarizine plus dihydroergocristine in 122 patients with cervical vertigo. Vestibulometric verification. *Current Therapeutic Research - Clinical and Experimental, 46*(6), 1196-1202.

Bittar, R., Alves, N. G. P., Bertoldo, C., Brugnera, C., & Oiticica, J. (2017). Efficacy of Carbon Microcoils in Relieving Cervicogenic Dizziness. *International Archives of Otorhinolaryngology, 21*(1), 4-7.

Bolton, P. S. (1992). Assessment of cervical vertigo. *Journal of Manipulative & Physiological Therapeutics, 15*(4), 267-268.

Borg-Stein, J., Rauch, S. D., & Krabak, B. (2001). Evaluation and management of cervicogenic dizziness. *Critical Reviews in Physical and Rehabilitation Medicine, 13*(2), 255-264.

Bracher, E. S., Almeida, C. I., Almeida, R. R., Duprat, A. C., & Bracher, C. B. (2000). A combined approach for the treatment of cervical vertigo. *Journal of Manipulative & Physiological Therapeutics, 23*(2), 96-100.

Brandt, T., & Bronstein, A. M. (2001). Cervical vertigo. *Journal of Neurology, Neurosurgery & Psychiatry, 71*(1), 8-12.

Brandt, T., & Huppert, D. (2016). A new type of cervical vertigo: Head motion-induced spells in acute neck pain. *Neurology, 86*(10), 974-975.

Chang, F., Li, Z., Xie, S., Liu, H., Wang, W. (2014). Vertigo-related cerebral blood flow changes on magnetic resonance imaging. *Spine, 39*(23), E1374-1379.

Chen, H., Shi, Z., Feng, H., Wang, R., Zhang, Y., Xie, J., . . . Zhou, H. (2015). The relationship between dizziness and cervical artery stenosis. *Neuroreport, 26*(18), 1112-1118. doi:10.1097/WNR.0000000000000478

Chen, R. X. (2002). Analysis of efficacy of traction plus computer middle frequency on cervical vertigo. *Chinese Journal of Clinical Rehabilitation, 6*(12), 1856-1857.

Endo, K., Suzuki, H., & Yamamoto, K. (2008). Consciously postural sway and cervical vertigo after whiplash injury. *Spine, 33*(16), E539-542.

Fitz-Ritson, D. (1991). Assessment of cervicogenic vertigo. *Journal of Manipulative & Physiological Therapeutics, 14*(8), 487-488.

Gu, C. L., Chen, X. D., Zhu, S. G., Lin, M. C., Liu, W. B., & Dai, J. Q. (2016). Effect of three vertigo-stopping needles on neurohumor of patients with cervical vertigo: a controlled trial. *World Journal of Acupuncture - Moxibustion, 26*(2), 25-30.

Hou, Z., Xu, S., Li, Q., Cai, L., Wu, W., Yu, H., & Chen, H. (2017). The Efficacy of Acupuncture for the Treatment of Cervical Vertigo: A Systematic Review and Meta-Analysis. *Evidence-Based Complementary & Alternative Medicine: eCAM, 2017*, 7597363.

Humphreys, B. K., Bolton, J., Peterson, C., & Wood, A. (2002). A cross-sectional study of the association between pain and disability in neck pain patients with dizziness of suspected cervical origin. *Journal of Whiplash and Related Disorders, 1*(2), 63-73.

Humphreys, B. K., & Peterson, C. (2013). Comparison of outcomes in neck pain patients with and without dizziness undergoing chiropractic treatment: a prospective cohort study with 6 month follow-up. *Chiropractic & manual therapies, 21*(1), 3.

Iwasa, H., Yagi, T., & Kamio, T. (1983). Diagnostic significance of neck vibration for the cervical vertigo. *Advances in Oto-Rhino-Laryngology, 30*, 268-270.

Jaroshevskyi, O. A., Payenok, O. S., & Logvinenko, A. V. (2017). Evalution of the effectiveness of multimodal approach to the management of cervical vertigo. *Wiadomosci Lekarskie, 70*(3), 571-573.

Johnston, J. L., Daye, P. M., & Thomson, G. T. (2017). Inaccurate Saccades and Enhanced Vestibulo-Ocular Reflex Suppression during Combined Eye-Head Movements in Patients with Chronic Neck Pain: Possible Implications for Cervical Vertigo. *Frontiers in neurology [electronic resource]. 8*, 23.

Jongkees, L. B. (1969). Cervical vertigo. *Laryngoscope, 79*(8), 1473-1484.

Krabak, B. J., Borg-Stein, J., & Oas, J. A. (2000). Chronic cervical myofascial pain syndrome: Improvement in dizziness and pain with a multidisciplinary rehabilitation program. A pilot study. *Journal of Back & Musculoskeletal Rehabilitation, 15*(2), 83-87.

Li, W., Tan, L., & Wang, S. Q. (2013). Sixty-five cases of cervical vertigo treated by needling cervical jiaj points in combination with acupoint pressing. *World Journal of Acupuncture - Moxibustion, 23*(2), 48-50.

Liu, X. M., Pan, F. M., Yong, Z. Y., Ba, Z. Y., Wang, S. J., Liu, Z., . . . Wu, D. S. (2017). Does the longus colli have an effect on cervical vertigo?: A retrospective study of 116 patients. *Medicine, 96*(12), e6365.

Ma, Y., Bu, H., Liu, Z., Jia, J. R., Li, X. Y., & Xu, Y. Q. (2015). Effect of acupuncture at Lieque ( LU 7) on vertebral-basilar artery hemodynamics in patients with cervical vertigo. *World Journal of Acupuncture - Moxibustion, 25*(2), 23-27.

Magnusson, M., & Malmstrom, E. M. (2016). The conundrum of cervicogenic dizziness. *Handbook of Clinical Neurology, 137*, 365-369.

Malmstrom, E. M., Karlberg, M., , M., er, A., Magnusson, M., & Moritz, U. (2007). Cervicogenic dizziness - musculoskeletal findings before and after treatment and long-term outcome. *Disability & Rehabilitation, 29*(15), 1193-1205.

Michels, T., Lehmann, N., & Moebus, S. (2007). Cervical vertigo - Cervical pain: An alternative and efficient treatment. *Journal of Alternative and Complementary Medicine, 13*(5), 513-518.

Minguez-Zuazo, A., , G., e-Alonso, M., Saiz, B. M., La Touche, R., & Lara, S. L. (2016). Therapeutic patient education and exercise therapy in patients with cervicogenic dizziness: a prospective case series clinical study. *Journal of Exercise Rehabilitation, 12*(3), 216-225.

Morinaka, S. (2009). Musculoskeletal diseases as a causal factor of cervical vertigo. *Auris, Nasus, Larynx, 36*(6), 649-654.

Moustafa, I. M., Diab, A. A., & Harrison, D. E. (2017). The effect of normalizing the sagittal cervical configuration on dizziness, neck pain, and cervicocephalic kinesthetic sensibility: a 1-year randomized controlled study. *European journal of physical & rehabilitation medicine., 53*(1), 57-71.

Norre, M. E., Forrez, G., Stevens, A., & Beckers, A. (1987). Cervical vertigo diagnosed by posturography? Preliminary report. *Acta Oto-Rhino-Laryngologica Belgica, 41*(4), 574-581.

Nwaorgu, O. G., Onakaoya, P. A., & Usman, M. A. (2003). Cervical vertigo and cervical spondylosis--a need for adequate evaluation. *Nigerian Journal of Medicine: Journal of the National Association of Resident Doctors of Nigeria, 12*(3), 140-144.

Okubo, Y. (1973). Panoramic tomography in cervical vertigo: (observation by orthopantomography). *Journal of Otolaryngology of Japan, 76*(7), I.

Reid, S. A., Callister, R., Katekar, M. G., & Rivett, D. A. (2014). Effects of cervical spine manual therapy on range of motion, head repositioning, and balance in participants with cervicogenic dizziness: a randomized controlled trial. *Archives of Physical Medicine & Rehabilitation, 95*(9), 1603-1612.

Reid, S. A., Callister, R., Snodgrass, S. J., Katekar, M. G., & Rivett, D. A. (2015). Manual therapy for cervicogenic dizziness: Long-term outcomes of a randomised trial. *Manual Therapy, 20*(1), 148-156.

Reid, S. A., Rivett, D. A., Katekar, M. G., & Callister, R. (2012). Efficacy of manual therapy treatments for people with cervicogenic dizziness and pain: protocol of a randomised controlled trial. *BMC Musculoskeletal Disorders, 13*, 201.

Ren, L., Guo, B., Zhang, J., Han, Z., Zhang, T., Bai, Q., & Zeng, Y. (2014). Mid-term efficacy of percutaneous laser disc decompression for treatment of cervical vertigo. *European journal of orthopaedic surgery & traumatologie, 24*, S153-158.

Reneker, J. C., Clay Moughiman, M., & Cook, C. E. (2015). The diagnostic utility of clinical tests for differentiating between cervicogenic and other causes of dizziness after a sports-related concussion: An international Delphi study. *Journal of Science & Medicine in Sport, 18*(4), 366-372.

Ryan, G. M., & Cope, S. (1955). Cervical vertigo. *Lancet, 269*(6905), 1355-1358.

Shum, G. L., Cinnamond, S., Hough, A. D., Craven, R., & Whittingham, W. (2017). Test-Retest Reliability of Measuring the Vertebral Arterial Blood Flow Velocity in People With Cervicogenic Dizziness. *Journal of Manipulative & Physiological Therapeutics, 40*(4), 255-262.

Tjell, C., & Rosenhall, U. (1998). Smooth pursuit neck torsion test: a specific test for cervical dizziness. *American Journal of Otology, 19*(1), 76-81.

van Leeuwen, R. B., & van der Zaag-Loonen, H. (2017). Dizziness and neck pain: a correct diagnosis is required before consulting a physiotherapist. *Acta Neurologica Belgica, 117*(1), 241-244.

Wakayama, T. (1973). Clinical study on the mechanism of the cervical vertigo. *Journal of Otolaryngology of Japan, 76*(7), I.

Wing, L. W., & Hargrave Wilson, W. (1974). Cervical vertigo. *Australian and New Zealand Journal of Surgery, 44*(3), 275-277.

Wu, X. P. (2002). Prospective study of three projects of treatment on chronic obstinate vertigo of cervical origin. *Chinese Journal of Clinical Rehabilitation, 6*(14), 2172-2173.

Xiaoxiang, Z. (2006). Jinger moxibustion for treatment of cervical vertigo --a report of 40 cases. *Journal of Traditional Chinese Medicine, 26*(1), 17-18.

Yacovino, D. A., & Hain, T. C. (2013). Clinical characteristics of cervicogenic-related dizziness and vertigo. *Seminars in Neurology, 33*(3), 244-255.

Yin, Y., Qin, X., Huang, R., Xu, J., Li, Y., & Yu, L. (2016). Musculoskeletal Ultrasound: A Novel Approach for Luschka's Joint and Vertebral Artery. *Medical Science Monitor, 22*, 99-106.

Zeng, X., Chen, S., Guan, C., Peng, Y., & Jiang, L. (2003). Effects of mental intervention on cervical vertigo. *Chinese Journal of Clinical Rehabilitation, 7*(1), 160.
